# Development and validation of the Transgender Adolescent Stress Survey-Dysphoria

**DOI:** 10.3389/fpsyg.2024.1448706

**Published:** 2024-09-19

**Authors:** Sheree M. Schrager, Jeremy T. Goldbach, Jules K. Wood, Rory P. O'Brien, Shannon L. Dunlap, Harmony Rhoades

**Affiliations:** ^1^Office of Graduate Studies and Research, California State University-Dominguez Hills, Carson, CA, United States; ^2^Sexuality, Health and Gender Center, George Warren Brown School of Social Work, Washington University in St Louis, Saint Louis, MO, United States; ^3^School of Social Work, University of Michigan, Ann Arbor, MI, United States; ^4^Luskin School of Public Affairs, University of California-Los Angeles, Los Angeles, CA, United States

**Keywords:** transgender, nonbinary, adolescents, gender, dysphoria, behavioral health

## Abstract

**Objective:**

Transgender and nonbinary adolescents (TNBA) may experience gender dysphoria arising from incongruities between their body and their gender. Prior dysphoria measures have largely focused on clinical diagnosis with little regard to comparability of forms for people assigned male or female at birth, overall psychometric performance, or applicability to nonbinary populations. This study develops and validates the Transgender Adolescent Stress Survey-Dysphoria (TASS-D), intended to address these gaps.

**Methods:**

The current study recruited a U.S. national sample of TNBA (*N* = 444, aged 12–17; 65.5% White, 9.5% Black, 9.5% Latine, 15.5% other ethnicity; 34.7% transmasculine, 17.3% transfeminine, 38.3% nonbinary, 9.5% agender). The item pool was developed from life history calendars, a modified Delphi process, and cognitive interviews with TNBA. Scale development included factor analysis, item response theory modeling, measurement invariance testing, and reliability analyses. Associations were examined between the TASS-D and existing measures of gender dysphoria (convergent validity), gender minority stress (divergent validity), and behavioral health outcomes (criterion validity).

**Results:**

TASS-D and its subscales (body distress and gender expression burden) were significantly and strongly associated with gender dysphoria; significantly but weakly associated with gender minority stress; and significantly associated with most indicators of psychological distress including depressive, anxiety, and posttraumatic stress symptoms, suicidal behaviors and nonsuicidal self-injury.

**Conclusions:**

The TASS-D is a reliable and valid measure of gender dysphoria for TNBA, offering notable benefits over existing measures: It is psychometrically sound, inclusive of all gender identities, and does not assume that respondents identify binarily or desire medical transition as a terminal goal.

## 1 Introduction

Compared to their cisgender peers, transgender and nonbinary adolescents (TNBA) report significantly higher rates of substance use, anxiety, depression, self-harm, and suicidality (Clark et al., 2014, 2018a; Eisenberg et al., 2017; Perez-Brumer et al., 2017; Toomey et al., 2018). Suicide risk is strikingly elevated among TNBA; for example, Toomey et al. (2018) found that transgender youth reported lifetime rates of suicide attempt almost three times higher than cisgender girls. In recent work (1F31HD091981) in Los Angeles, 45% of transgender youth participants reported having a previous suicide attempt. Recent Youth Risk Behavior Survey data demonstrated heightened risk among transgender youth of exposure to stressors and mental health outcomes including hopelessness, bullying, forced sexual intercourse, and being threatened with a weapon at school (Johns et al., 2019).

Although TNBA are frequently grouped with cisgender sexual minority adolescents into one collective category (i.e., LGBT), TNBA repeatedly exhibit more pronounced disparities. For instance, 23% of cisgender sexual minority youth across the United States report an attempted suicide at some point in their lives, compared with 32.3% of TNBA (Kuper et al., 2018). Analogous trends are evident in the reporting of anxiety symptoms (Bockting et al., 2013; Bostwick et al., 2009), depression (Bockting et al., 2013; Russell and Fish, 2016), substance abuse (Reisner et al., 2015; Watson et al., 2018), and PTSD (Russell and Fish, 2016; Wharton, 2007). Indeed, this mounting evidence demands a more nuanced understanding of factors that might drive these and other health disparities for TNBA.

One particular type of stress that likely contributes to behavioral health outcomes among TNBA is gender dysphoria. Although controversial, gender dysphoria is defined by the DSM-5 (American Psychiatric Association, 2013) as: (a) “a marked incongruence between the gender assigned at birth and experienced/expressed gender”; and (b) either “clinically significant distress” or “impairment in social, school/occupational, or other areas of functioning.” Although the updated language regarding gender dysphoria captures both incongruence and social aspects of distress, the criteria do not distinguish among possible causes of the supposed impairment, such as social stigma. Notably, although the definitional criteria for gender dysphoria require the experience of either distress or functional impairment, research has demonstrated that not all transgender and nonbinary people experience such distress, even if they do experience incongruence between their appearance and gender identity (Galupo et al., 2021). This study, therefore, considered incongruence and dysphoria to be related but distinct experiences, each meriting measurement. Although research has broadly supported the fact that gender dysphoria is a significant source of stress demanding intervention (Olson et al., 2015; Peterson et al., 2017), less is known about the many ways TNBA experience gender dysphoria during adolescence and how such experiences may be related to psychological distress.

For TNBA, puberty brings many social norms and pressures attached to developing bodies (Nuttbrock et al., 2010; Olson et al., 2011; Yunger et al., 2004), and the development of secondary sex characteristics that are incongruent with felt gender during adolescence (Burgess, 1999) can be associated with mental health concerns (de Vries et al., 2011; Nuttbrock et al., 2010; Olson et al., 2011; Yunger et al., 2004). Yet despite the need for psychometrically strong adolescent-appropriate dysphoria measures that can support etiological research, clinical assessment, and the development of interventions to support TNBA, few options currently exist. A particularly critical concern regarding existing measures is the ability to differentiate the stress of dysphoria from stresses associated with the internalization of discrimination and stigma, as conceptualized in minority stress theory (Meyer, 2003; Testa et al., 2015). Thus, existing measures such as the Utrecht Gender Dysphoria Scale (UGDS) (Cohen-Kettenis and Van Goozen, 1997) conflate dysphoria (e.g., “I hate my breasts”) with minority stress (e.g., “Every time someone treats me like a girl or woman, I feel hurt”), likely mischaracterizing both. Similarly, existing measures focus near exclusively on assessing DSM criteria and do little to capture the socioemotional stress of gender dysphoria that may persist independent of physical transition. This concern has been illustrated by trans youth in a recent study (Galupo and Pulice-Farrow, 2019) regarding their subjective experiences of completing the Gender Identity/Gender Dysphoria Questionnaire for Adolescents and Adults (GIDYQ-AA; Deogracias et al., 2007) and UGDS, with only 54% of respondents stating that their experience was captured by these measures.

One additional major limitation of existing dysphoria measures is their inherent promotion of a binary representation of gender. TNBA may identify with either a binary (trans boy or trans girl; girl or boy) or nonbinary gender identity such as genderqueer, gender fluid, nonbinary, neither male or female, or both male and female (Frazer and Dumont, 2016; Gates, 2011). Galupo et al. (2021), in a study of agender and nonbinary adults, noted that traditional concepts of dysphoria related to “rejecting one set of (binary) features in favor of the ‘opposite' was inadequate” (p. 108) and that the use of hormones in this population may simultaneously relieve some types of body distress while exacerbating others by shifting body parts to be more masculine or feminine than desired. Thus, the gender dysphoria experienced by agender and nonbinary individuals may be felt or expressed differently than for binary-identified transgender youth and adults; may be largely misunderstood across social contexts, including medical settings (Galupo et al., 2021); and merits more intentional assessment than possible with currently available measures. Therefore, the present study sought to develop and validate a measure of dysphoria that could be used with TNBA.

## 2 Preliminary studies

### 2.1 Qualitative study

As a foundation for developing a testable set of items, we employed a life history calendar approach to conduct separate and simultaneous qualitative interviews with 20 TNBA–parent dyads to explore dyadic perspectives of adolescent minority stress, gender dysphoria, parent support, and adolescent gender-affirmation processes during childhood and adolescence. Adolescents were between the ages of 12 and 17 and had initiated puberty blockers or gender affirming hormones or both during the 12 months prior to the interview. Three coders independently determined codes to attach to text fragments representing descriptions of gender dysphoria, using axial coding to place individual fragments into conceptual domains and convert them into short, salient statements that would be understood by 12- to 17-year-old TNBA. These statements were reviewed by the first two authors for conceptual centrality to the dysphoria construct. An initial candidate list of 32 dysphoria items was developed and shared with an expert panel as part of a modified Delphi process.

### 2.2 Modified Delphi study

A panel of six experts in TNBA medicine, behavioral health, gender dysphoria, and minority stress was convened to participate in a modified expert panel study following the RAND/UCLA Appropriateness Method (Fitch et al., 2001), also known as a modified Delphi process, adapted for use with socio-behavioral measure development (Schrager and Goldbach, 2017). The research team held a 60-min training session with the expert panelists, who then had 3 weeks to rate the 32 candidate dysphoria items for feasibility (i.e., whether a TNBA could reasonably be expected to read, understand, and accurately self-report their experiences with that item) and validity (i.e., whether the item truly represented the psychological construct of dysphoria). Validity and feasibility for each item were rated on a 9-point ordinal scale (1 = *low*, 9 = *high*), and scores were examined for consensus to accept, consensus to reject, or discrepant opinions by the panel. After the first round of ratings, the expert panel did not reject any dysphoria items, but all 32 items were found to be discrepant and required additional discussion.

Three panel meetings were held via Zoom for 15 h across 3 days. All dysphoria items were discussed, and six items were rejected during the panel discussion. The expert panel had another 2 weeks to provide second-round ratings on the remaining 26 dysphoria items. After the second round of ratings, 16 items deemed valid and feasible by the expert panel were retained as the initial dysphoria measure. An additional four items, though rated with discrepancy by the expert panel, were determined to be sufficiently central to the dysphoria construct (e.g., “My body does not match my gender identity”) that the study team elected to include them as optional items in the cognitive interview phase, for a total of 20 items.

### 2.3 Cognitive interviews

Fourteen TNBA aged 12–17 were recruited via contacts at several transgender adolescent and family community-based programs to participate in cognitive interviews. For each proposed dysphoria item, youth were asked “Is the statement clear or understandable, or confusing in any way?”; “Were any of the words offensive or difficult to understand?” (if so, follow-up questions asked for further explanation and recommendations for rewording); and “Is this something you could see happening to somebody like you?” Following the cognitive interviews, 11 items were retained without changes, seven items were lightly reworded for clarity, two items were deleted, and two new items, related to body hair and feeling like an outsider in one's body, were added. This revised 20-item candidate dysphoria measure was included in the main study.

## 3 Methods

### 3.1 Participants and procedures

Participants in the main study were recruited and enrolled in two phases. Starting in January 2022, targeted advertisements were shown on social media platforms, specifically Instagram and YouTube. However, due to a change in Instagram advertising policies around this time, we could not target our audience on this platform using interest keywords. To narrow the scope of the recruitment campaign, advertisements were targeted by age, geographic region (West, Midwest, Southwest, Southeast, Northeast), and urbanicity using the 2020 Rural-Urban Commuting Area taxonomy for coding urbanicity by ZIP code (Cromartie, 2020). The advertisements promoted links to a subject pool screener where youth entered their demographics and contact information and were informed that they would be contacted if they were eligible for future studies. Youth were eligible to participate in the present study if they were 12–17 years old; resided in the United States or a U.S. territory; responded “yes” to at least one of three questions assessing if they were transgender, nonbinary, or genderqueer or if they did not identify with one of these three labels, reported any gender identity that did not match their sex assigned at birth; and were willing and able to provide assent to participate in the study.

After reviewing demographic information provided in the subject pool screener, the study team individually contacted eligible youth via the contact information they provided to invite them to participate in the initial study survey, which was an online survey deployed in Qualtrics. This survey initially asked demographic questions to verify study eligibility before advancing to the assent information screen. After providing assent, participants first completed a battery of 89 newly developed items (including the 20 proposed dysphoria items), followed by validation measures. However, during pilot testing, the complete survey was determined to be too long and burdensome for adolescents. Thus, the validation measures were divided across three shorter survey versions. When invited to participate in the study, respondents were randomly assigned to receive one of the three survey versions, each containing the newly developed dysphoria measure along with a subset of validation measures. In total, 444 participants completed one of the three primary survey versions between March and May 2022.

To ensure data quality, participants who completed the survey in an improbably short timespan (…) or failed to complete at least three of four attention-control items correctly (e.g., “Please select ‘Decline to Answer”') were excluded from the data prior to analysis (Robinson-Cimpian, 2014; Bauermeister et al., 2012). Participants who attempted to gain re-entry to the survey to receive additional compensation, such as via multiple attempts from the same IP address or providing contact information from an existing participant, were also excluded from analysis to prevent duplicate participation (Grey et al., 2015; Teitcher et al., 2015). Participants who successfully completed the baseline survey were invited to recruit additional participants from their personal networks in a respondent-driven sampling process (Heckathorn, 1997). Participants received an email containing three unique survey links and language prompts to encourage peers to participate. For each eligible participant who completed the baseline survey, the participant who referred that individual received an additional $10 online gift card.

**Figure 1:**
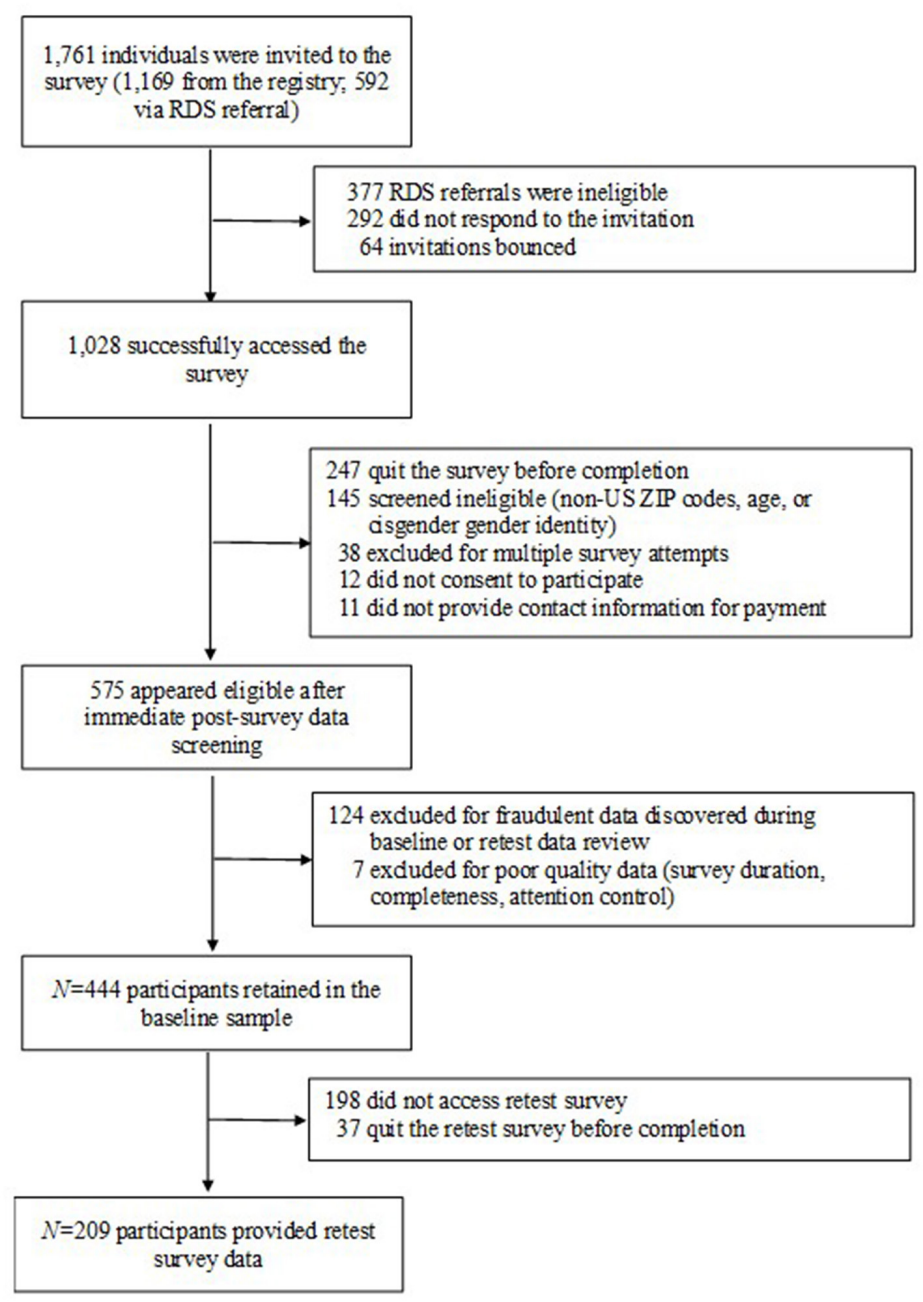
CONSORT flow diagram for study enrollment (final *N* = 444 for baseline, *N* = 209 for retest).

Two weeks after participants took the initial survey, they were contacted again with a request to complete a retest survey of only the newly developed measures and provide sufficient demographic information to verify accurate data file matching. Participants received a $25 online gift card for completion of the baseline survey and a $10 online gift card for completion of the retest. Of the 444 participants who completed the initial survey, 246 (55.4%) also participated in the retest survey, which was completed between April and June 2022 based on each respondent's baseline participation date. The full CONSORT diagram illustrating participant recruitment and retention through the test–retest period is presented in Figure 1.

### 3.2 Measures

All participants completed the following measures:

#### 3.2.1 Gender dysphoria

**Figure 2:**
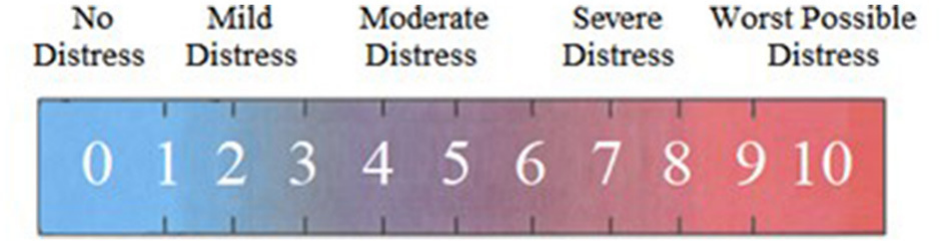
Visual analog scale (VAS) for dysphoria-related distress.

The key measure in the current study, the candidate dysphoria measure, consisted of 20 statements describing gender dysphoria. Participants were initially provided with the 20 statements and instructed to “choose ‘yes' if the statement describes something that you experience or ‘no' if it does not reflect your personal experience.” “Yes” responses were coded 1 and “no” responses were coded 0 for analysis. For each item endorsed, participants were subsequently asked to select “the number that best describes how much distress you feel from that experience” by moving a slider bar along a visual analog thermometer ranging from 0 (*no distress*) to 10 (*worst possible distress*; see Figure 2). For binary dysphoria items that were not endorsed, their corresponding distress item was coded as 0, reflecting no distress.

#### 3.2.2 Eligibility-related demographics

To verify eligibility to participate, participants were again asked to report their age, country of residence (recoded as 1 = *United States*, 0 = *all others*); five-digit ZIP code; sex assigned at birth (0 = *female*, 1 = *male*); and binary indicators (1 = *yes*, 0 = *no*) regarding whether they identify as transgender, nonbinary, genderqueer, or a self-reported gender identity different from their sex assigned at birth (Table 1).

**Table 1:** Demographic descriptive statistics.

**Variable**	***N* (%)**
Race/Ethnicity
White	291 (65.5)
Black	42 (9.5)
Hispanic/Latinx	42 (9.5)
Other	69 (15.5)
US region
Northeast	69 (15.5)
Southeast	100 (22.5)
Midwest	90 (20.3)
Southwest	57 (12.8)
West	123 (27.7)
Missing	5 (1.1)
Urbanicity
Urban	367 (82.7)
Rural	72 (16.2)
Missing	5 (1.1)
Age group
12–14	64 (14.4)
15	107 (24.1)
16	163 (36.7)
17	110 (24.8)
Pubertal development
Minimal devel., no blocker/hormones	82 (18.5)
Significant level, no blockers/hormones	252 (56.7)
Experience with blockers/hormones	83 (18.7)
Missing	27 (6.1)
Sex assigned at birth
Female	327 (73.6)
Male	117 (26.4)
Sexual identity
Gay/Lesbian	116 (26.1)
Bisexual/pansexual	229 (51.6)
Asexual	56 (12.6)
Queer/unlabeled/other	43 (9.7)
Gender identity
Transmasculine	154 (34.7)
Transfeminine	77 (17.3)
Nonbinary	170 (38.3)
Agender	42 (9.5)
Missing	1 (0.2)

#### 3.2.3 Other demographics

Although not used to determine eligibility, all participants were also asked to report their sexual identity or orientation, race and ethnicity, current school enrollment (binary), highest grade completed, a 4-point item assessing family socioeconomic status (“doesn't meet basic needs” to “live comfortably”), prior and current employment, current living situation, experiences of homelessness (assessed with one item, “Have you ever had to spend the night somewhere other than your home because you had nowhere else to stay?” with positive responses incurring follow-up items asking about experiences with different types of shelter), personal and family religion, binary items assessing prior and current use of pubertal blockers or hormone replacement therapy, and five items assessing pubertal development.

#### 3.2.4 Criterion validity measures

Behavioral health outcomes used to establish criterion validity included the Center for Epidemiological Studies Depression Scale-4 (CES-D-4; Melchior et al., 1993), an abbreviated version of the revised full version containing four self-report items that measure past-week depressive symptoms (α = 0.83). Anxiety symptoms were measured using the seven-item Generalized Anxiety Disorder-7 (GAD-7), which assesses how often a person has been bothered by certain problems during the past 2 weeks (α = 0.88). The six-item PTSD Checklist-Civilian (PCL-C-6; Lang et al., 2012) is an abbreviated 6-item version of the 17-item version that assesses symptoms of PTSD (α = 0.82). Suicidality was assessed with five items from the Youth Risk Behavior Survey (Johns et al., 2019) and recoded into binary indicators (1 = *yes*, 0 = *no*) of past 12-month suicidal ideation, suicide attempt, and nonsuicidal self-injury. Finally, lifetime and past-30-day use of alcohol, marijuana, and other drugs were measured with the corresponding substance use items from the Youth Risk Behavior Survey.

#### 3.2.5 Convergent and divergent validity measures

During pilot testing of the full-length survey with the study team, the complete survey was determined to be too long and burdensome for adolescents. Thus, the validation measures were divided across three shorter survey versions. At the time they were invited to participate in the study, respondents were randomly assigned to receive one of the three survey versions, each of which contained a subset of validation measures, as follows.

#### 3.2.6 Survey A

The first survey version included two convergent validation measures: the 18-item UGDS-Gender Spectrum (UGDS-GS; McGuire et al., 2020, α = 0.90) and the GIDYQ-AA (Deogracias et al., 2007), which has 27-item versions intended for participants assigned female at birth (GIDYQ-FAB, α = 0.82) or assigned male at birth (GIDYQ-MAB, α = 0.80). Scoring for these measures follows the scoring procedures in the original citations.

#### 3.2.7 Survey B

The second version contained the 60-item Gender Minority Stress and Resilience Measure (GMSR; Hidalgo et al., 2019), used for divergent validation. The GMSR does not yield a single total score but rather nine subscale scores reflecting gender-related discrimination, rejection, and victimization; gender identity nonaffirmation; internalized transphobia; negative expectations for the future; nondisclosure of gender identity or history; pride; and community connectedness (α's = 0.69–0.89).

#### 3.2.8 Survey C

The third and final survey version included the original UGDS (Cohen-Kettenis and Van Goozen, 1997), which includes 12-item versions intended for participants assigned female at birth (UGDS-F, α = 0.88) or assigned male at birth (UGDS-M, α = 0.88), to assess convergent validity. Survey version C also included the Sexual Minority Adolescent Stress Inventory (Goldbach et al., 2017; Schrager et al., 2018) to establish divergent validity. The inventory includes 54 items across 10 primary domains assessing lifetime and 30-day LGBTQ minority stress, with an additional 10 items assessing work-related minority stressors among participants who were currently or previously employed (α = 0.92 for lifetime scores; α = 0.88 for past 30-day scores).

### 3.3 TASS-D analytic plan

We employed psychometric methods to construct and validate the final TASS-D scale from the 20 candidate items. First, we examined ratings of distress for items participants rated as sources of dysphoria to check that distress showed adequate variability between persons. Having found enough variability in dysphoric distress to justify distress as a relatively continuous construct, we recoded items not rated as sources of dysphoria to represent distress ratings of zero. To begin to identify the structure underlying distress ratings, we conducted exploratory factor analysis (EFA) with the 20 candidate items to identify the approximate number of factors to adequately explain our data. After identifying the optimal number of factors and structure of factor loadings, we conducted confirmatory analyses of the structure and fit of our models. At this stage, we also conducted item response theory (IRT) analyses of the subscales and total scale, eliminating items that showed weak discriminability between levels of the underlying latent trait (i.e., dysphoria).

Having identified the structure of the TASS-D, we conducted measurement invariance analyses to establish that the proposed scale operated in the same way across levels of various demographic variables. The variables assessed for measurement invariance were race and ethnicity (White, Black, Hispanic or Latinx, or other); U.S. region (West, Southwest, Midwest, Southeast, or Northeast); urbanicity (urban or rural); age group (12–14, 15, 16, or 17), pubertal development status or experience with puberty blockers and hormones (categorized as having experience with blockers or hormones, having minimal pubertal development and no blockers or hormones, or having significant body changes and no blockers or hormones); sexual identity (gay or lesbian, bisexual or pansexual, asexual, or queer or other); sex assigned at birth (male or female); and gender identity (transmasculine, transfeminine, nonbinary, or agender). Measurement invariance analyses tested configural invariance (same pattern of factor loadings across groups), metric invariance (fixed factor loadings across groups), and scalar invariance (fixed item means across groups). Items that presented substantial noninvariance at this stage were candidates for removal.

Finally, with a well-fitting and consistent scale, we conducted a series of analyses to test the reliability and validity of our new scale. Reliability was assessed using Cronbach's alpha to test the internal consistency of the full scale and subscales. To assess convergent validity, we analyzed the association between the TASS-D and other existing measures of gender dysphoria (e.g., UGDS, UGDS-GS, and GIDYQ-AA). To assess divergent validity, we examined associations between the TASS-D and a measure of gender minority stress (GMSR). Finally, we sought to establish criterion validity of the TASS-D by assessing its hypothesized relationship with measures of mental health and substance use, with the expectation that higher levels of gender dysphoric distress would be associated with higher depression, anxiety, and PTSD symptoms and higher odds of substance use.

## 4 Results

### 4.1 TASS-D structural analyses

A principal components analysis indicated that a two- or three-factor solution would be adequate to explain our data. The model was fit using the *prcomp*() function in R (R Core Team, 2022). The number of ideal factors was determined by a visual inspection of the scree plot alongside the number of principal components with eigenvalues >1, both of which indicated that a maximum of three factors would efficiently explain most of the variance in the data.

Two- and three-factor EFA models were fit using oblique promax rotation with the fa() function from the *psych* package in R (Revelle, 2023). The factor loadings and correlations for both models are given in Table 2. Given that factor correlations were substantial (and theoretically, dimensions of dysphoria are likely interrelated), the use of oblique rotation was supported. Comparing the two- and three-factor solutions, the third factor that emerged in the three-factor solution contained only two items related to body hair and facial hair. These were determined to not constitute a theoretically relevant latent variable on their own, so we selected the two-factor solution. Items with factor loadings >0.50 were moved to the confirmatory factor analysis stage. Based on the item loadings of the two-factor solution, we identified two initial subscales: one for body-related dysphoria (Items 2–4, 7–10, 17, and 18) and one for gender expression-related burden (Items 14–16, 19, and 20).

**Table 2:** EFA results for 2- and 3-factor models of dysphoric distress.

	**2-factor EFA**		**3-factor EFA**		
	**Factor 1**	**Factor 2**	**Factor 1**	**Factor 2**	**Factor 3**
**Factor loadings**					
Item 1: My voice does not match my gender	0.40	0.28	0.37	0.27	0.09
Item 2: My genitals do not match my gender identity	**0.78**	−0.21	0.71	−0.21	0.12
Item 3: The size of my chest does not match my gender identity	**0.55**	0.24	**0.64**	0.19	−0.07
Item 4: My facial hair does not match my gender	**0.51**	−0.03	0.06	0.02	**0.84**
Item 5: My body hair does not match my gender	0.42	−0.04	−0.01	0.03	**0.76**
Item 6: Some of my body parts do not match my gender identity	**0.68**	0.14	**0.74**	0.10	−0.03
Item 7: The way my body experiences sexual arousal feels wrong to me	**0.60**	−0.19	**0.61**	−0.21	0.01
Item 8: My body does not match my gender identity	**0.53**	0.34	**0.59**	0.30	−0.03
Item 9: My body is developing in ways that feel wrong for my gender identity	**0.73**	0.10	**0.74**	0.06	0.03
Item 10: I wish my body had gone through a puberty that matched my gender instead of my sex assigned at birth	**0.64**	0.16	**0.57**	0.15	0.15
Item 11: I am uncomfortable looking at photos of myself that remind me of my sex assigned at birth	0.44	0.13	0.40	0.12	0.09
Item 12: I feel like an outsider in my own body	0.30	0.45	0.38	0.40	−0.07
Item 13: My height will never match my gender	0.11	0.32	0.16	0.30	−0.04
Item 14: My body will never fully match my gender	0.15	**0.65**	0.19	**0.62**	−0.01
Item 15: Making my gender expression match my gender will be a burden for the rest of my life	−0.18	**0.92**	−0.26	**0.95**	0.14
Item 16: I worry I will never be seen as my gender identity	−0.10	**0.77**	−0.02	**0.73**	−0.07
Item 17: I try to hide parts of my body because they do not match my gender identity	**0.62**	0.17	**0.72**	0.11	−0.09
Item 18: I try to avoid seeing parts of my body that do not match my gender identity	**0.62**	0.07	**0.73**	0.01	−0.09
Item 19: Making my appearance match my gender identity is exhausting	−0.10	**0.83**	−0.09	**0.81**	0.04
Item 20: It takes a lot of effort to express my gender identity	−0.11	**0.82**	−0.08	**0.80**	<0.01
**Factor correlations**					
Factor 1	1	0.68	1	0.68	0.42
Factor 2	0.68	1	0.68	1	0.30
Factor 3	–	–	0.42	0.30	1

These initial subscales were included in structural models within an IRT framework to assess the item response characteristics of individual items and conduct measurement invariance testing.

### 4.2 IRT analyses

The IRT characteristics of each item were analyzed with the Graded Response Model (GRM), an IRT method for ordinal-type data (Samejima, 1969). The GRM estimates two features of item responses: item *extremity*, which is the level of the latent trait at which a participant has a 50% chance of endorsing the next-highest response option of an ordinal variable and item *discriminability*, which is a measure of how well different response options discriminate between different levels of the underlying latent trait. Extremity estimates do not assume equal spacing between ordinal response options, and instead allow free estimation of corresponding latent trait levels measured by different responses. To fit the models, we used the *grm* function from the *ltm* package in R (Rizopoulos, 2006). In the models we fit, we allowed discriminability to be estimated freely for each item, so we could identify items with poor discriminability. Because the GRM assumes unidimensionality, we fit them separately for each subscale identified by EFA.

#### 4.2.1 Body-related distress subscale

**Figure 3:**
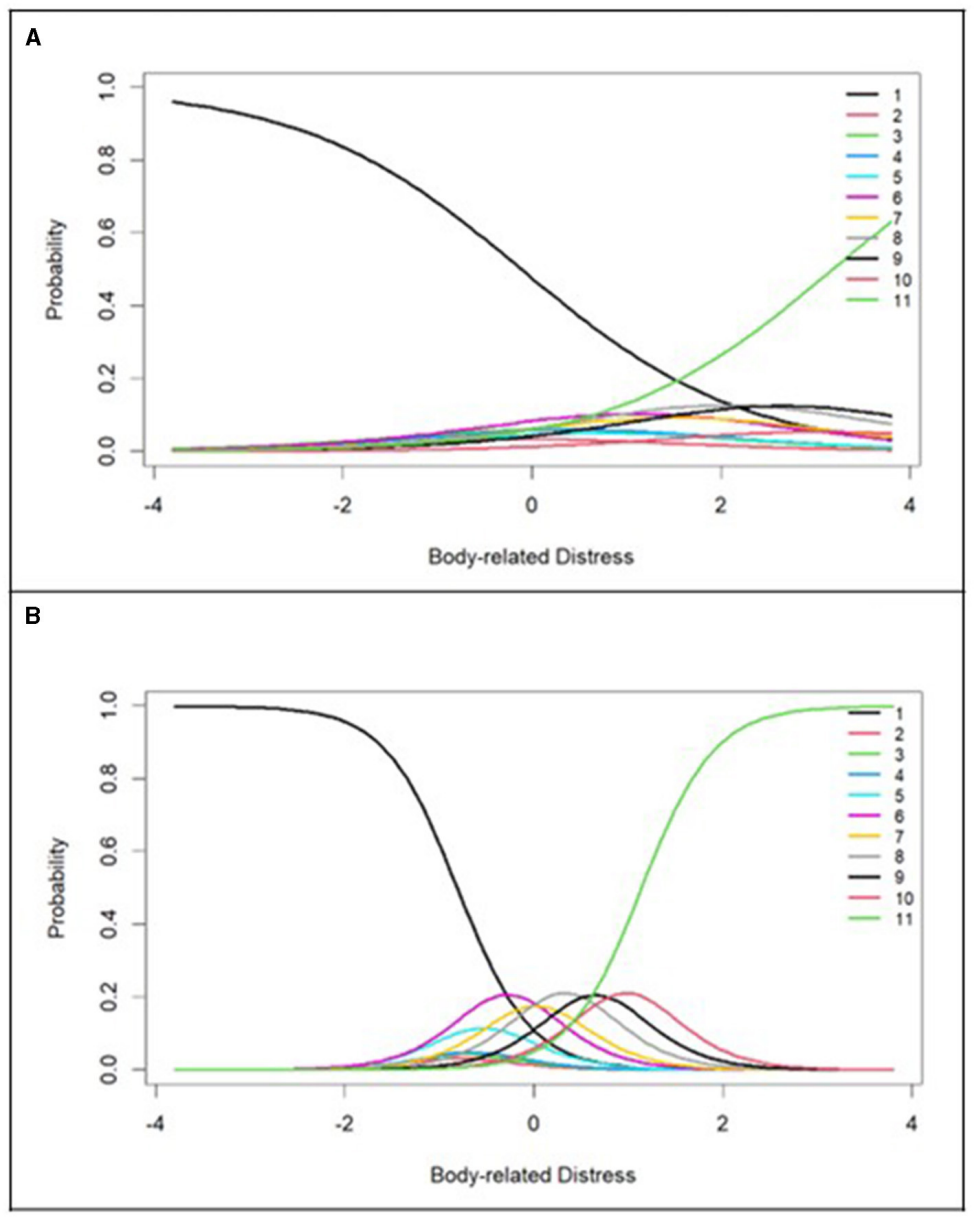
Select Item Response Characteristic Curves from GRM. **(A)** Item 4 (My facial hair does not match my gender identity). **(B)** Item 9 (My body is developing in ways that feel wrong for my gender identity).

This subscale initially consisted of nine items. Minimum extremity estimates ranged from −0.80 to −1.19. Maximum extremity estimates ranged from 0.93 to 3.18. Discriminability estimates ranged from 0.86 to 2.60. Full extremity and discriminability estimates are available on request. The item response characteristic curves (IRCCs) for the item with lowest discriminability (Item 4: “My facial hair does not match my gender identity”) are shown in Figure 3A. Poor discriminability is evident in the figure, with the lowest response option (0 = *no distress*) and highest response option (10 = *the most distress possible*) having the greatest probability of endorsement along all levels of the latent trait and the point at which these IRCCs cross (indicating a 50–50 chance of endorsing no distress or maximal distress caused by body hair) occurs at a relatively high level of the standardized latent trait (~1.5). Compared to the IRCCs of the item with the highest discriminability of this subscale (Item 9: “My body is developing in ways that feel wrong for my gender identity”), shown in Figure 3B, the difference in discriminability is clear. The IRCCs for Item 9 show a clearer separation in probability of endorsement between the levels of latent body-related distress. Due to its poor discriminability properties, Item 4 was dropped from this subscale.

#### 4.2.2 Gender expression burden subscale

This subscale consisted of five items. Minimum extremity estimates ranged from −0.56 to −1.27. Maximum extremity estimates ranged from 0.83 to 1.35. Discriminability parameters were acceptable for all items, ranging from 1.99 to 3.11. Full GRM parameter estimates are available on request. No items were excluded from the subscale based on this analysis, because the discriminability of all items was deemed sufficient.

### 4.3 Measurement invariance testing

The final stage of establishing the structure of the TASS-D was to conduct measurement invariance analyses to ensure that the scale and subscales functioned similarly across various demographic groups. Factor models were fit using the *cfa* function from the *lavaan* package in R (Rosseel, 2012). We assessed measurement invariance by U.S. region, urbanicity, race and ethnicity, age group, pubertal development status and experience with puberty blockers and hormones, sexual identity, sex assigned at birth, and gender identity in three increasingly restricted forms. First, we tested configural invariance, where the patterns of factor loadings were the same across groups, but the values of factor loadings were freely estimated. Configural invariance was determined by adequate fit statistics of the overall model, meeting at least one of the commonly accepted benchmarks of CFI > 0.90 TLI > 0.90, and RMSEA <0.10. Second, in metric invariance tests, factor loadings were constrained to be equal across groups. Finally, scalar invariance tests constrained item means (intercepts in the factor model) to be equal across groups. The fit of scalar and metric invariance models was compared with the fit of the configural invariance model using a likelihood ratio test (LRT) of nested models. If the LRT was not significant, the fit of the more constrained model was not worse than the less-constrained model and the stricter measurement invariance model was considered valid. If the chi-square statistic from the LRT was significant, then the stricter measurement invariance model had a worse fit than the less constrained model. If stricter measurement invariance failed at the scalar or metric stage, then we examined the modification indices, freeing factor loading or item intercept equality constraints to form partial invariance models until the fit of the model passed the LRT.

The version of the survey following the IRT analyses showed issues with metric invariance across gender identities and stages of pubertal development or experience with blockers and hormone therapy. Inspection of the modification indices indicated that Item 10 (“I wish that my body had gone through puberty that matched my gender instead of my sex assigned at birth”) was the source of this measurement noninvariance. At this stage, we elected to exclude Item 10 from the body-related dysphoric distress subscale. The following measurement invariance results are from the final scale, composed of a seven-item body dysphoria and a five-item gender expression burden subscale.

#### 4.3.1 Regionality

U.S. regions were divided into the West, Southwest, Midwest, Northeast and Southeast. Configural invariance across regions was supported with adequate, if borderline, fit metrics (CFI = 0.897, TLI = 0.871, RMSEA = 0.119). Metric invariance did not show decrement in fit, Δχ^2^(40) = 46.19, *p* = 0.23, CFI = 0.894, TLI = 0.886, RMSEA = 112, nor did scalar invariance, Δχ^2^(40) = 36.45, *p* = 0.63, CFI = 0.896, TLI = 0.900, RMSEA = 0.105.

#### 4.3.2 Urbanicity

Urbanicity was characterized as urban or rural. Configural invariance between groups was supported with good fit (CFI = 0.924, TLI = 0.905, RMSEA = 0.100). Metric invariance was supported as not worse in fit than configural invariance, Δχ^2^(10) = 12.86, *p* = 0.23, CFI = 0.923, TLI = 0.912, RMSEA = 0.096, as was scalar invariance, Δχ^2^(10) = 11.31, *p* = 0.33, CFI = 0.922, TLI = 0.919, RMSEA = 0.092.

#### 4.3.3 Race and ethnicity

Race and ethnicity was measured as White, Black, Hispanic or Latinx, and other. Configural invariance was supported as well-fitting by one fit statistic (CFI = 0.900) and adequate but borderline by the two others (TLI = 0.875, RMSEA = 0.115). Metric invariance was supported by the LRT, Δχ^2^(30) = 28.89, *p* = 0.52, CFI = 0.900, TLI = 0.891, RMSEA = 0.107, as was scalar invariance, Δχ^2^(30) = 33.15, *p* = 0.32, CFI = 0.899, TLI = 0.902, RMSEA = 0.101.

#### 4.3.4 Age group

Age group was divided into ages 12–14, 15, 16, and 17. Configural invariance was supported by CFI = 0.905, with acceptable TLI = 0.881 and RMSEA = 0.113. Metric invariance was supported by the LRT, Δχ^2^(30) = 15.82, *p* = 0.98, and adequate fit metrics (CFI = 0.910, TLI = 0.902, RMSEA = 0.103). Scalar invariance was also supported, Δχ^2^(30) = 36.79, *p* = 0.18, CFI = 0.908, TLI = 0.910, RMSEA = 0.098.

#### 4.3.5 Pubertal development and experience with blockers or hormones

Our pubertal development variable was divided into three categories: little pubertal development and no experience with blockers or hormones, significant pubertal development and no experience with blockers or hormones, and any stage of pubertal development with experience with blockers or hormones. Configural invariance was supported across pubertal development groups (CFI = 0.900, TLI = 0.875, RMSEA = 0.115), as was metric invariance, Δχ^2^(20) = 26.87, *p* = 0.14, CFI = 0.905, TLI = 0.896, RMSEA = 0.105. Pure scalar invariance showed a decrement in fit, compared to the less-constrained models, Δχ^2^(20) = 99.81, *p* < 0.001. Through iterative freeing of intercept equality constraints, we found that partial scalar invariance fit well with the intercepts of Items 2, 9, 15, and 16 allowed to vary between the groups of this variable, Δχ^2^(12) = 17.70, *p* = 0.12, CFI = 0.89, TLI = 0.89, RMSEA = 0.108.

#### 4.3.6 Sexual identity

Sexual identity was characterized as bisexual or pansexual, lesbian or gay, asexual, or queer or other. Configural invariance was supported across sexual identities (CFI = 0.906, TLI = 0.883, RMSEA = 0.112). Metric invariance was also supported, Δχ^2^(30) = 33.39, *p* = 0.31, CFI = 0.905, TLI = 0.896, RMSEA = 0.105; however, total scalar invariance was not, Δχ^2^(30) = 49.97, *p* = 0.01. Freeing the intercept of Item 14 on the body distress factor resulted in adequate partial scalar invariance, Δχ^2^(27) = 39.78, *p* = 0.053, CFI = 0.900, TLI = 0.902, RMSEA = 0.102.

#### 4.3.7 Sex assigned at birth

Sex assigned at birth was categorized as male or female. Configural invariance was supported across sex assigned at birth (CFI = 0.919, TLI = 0.899, RMSEA = 0.101). Metric invariance was also supported, Δχ^2^(10) = 8.10, *p* = 0.62, CFI = 0.920, TLI = 0.909, RMSEA = 0.096. However, total scalar invariance was not supported, Δχ^2^(10) = 26.19, *p* = 0.003. Freeing the intercept of Item 2 (“My genitals do not match my gender”) across groups resulted in adequate fit of partial scalar invariance, Δχ^2^(9) = 15.66, *p* = 0.07, CFI = 0.917, TLI = 0.913, RMSEA = 0.094.

#### 4.3.8 Gender identity

Gender identity was characterized as transfeminine, transmasculine, agender, or nonbinary or other. Configural invariance was supported with adequate fit (CFI = 0.905, TLI = 0.896, RMSEA = 0.105). Metric invariance was supported across gender identity groups, Δχ^2^(30) = 40.85, *p* = 0.09, CFI = 0.89, TLI = 0.894, RMSEA = 0.105. Total scalar invariance was not supported, however, Δχ^2^(30) = 69.00, *p* < 0.001. By freeing the intercept equality constraint for Item 2 (“My genitals do not match my gender”) and Item 16 (“I worry I will never be seen as my gender”), we achieved acceptable partial scalar measurement invariance, Δχ^2^(24) = 30.17, *p* = 0.18, CFI = 0.903, TLI = 0.904, RMSEA = 0.099.

### 4.4 Scoring

The final TASS-D included 12 items (full item text and subscales can be found in Appendix A). Scores for the total TASS-D scale and its two subscales were calculated by summing distress ratings for a maximum overall score of 120 on the total scale (70 and 50 for the body distress and gender expression burden subscales, respectively). Missing values were mean-imputed if a participant was missing two or fewer item responses. If they were missing more than two items, the scale score was considered missing overall. This resulted in 433 participants having non-missing total scale scores for the validation analyses. The mean TASS-D score was 64.65 (*SD* = 30.76).

Subscale scores were calculated with a similar mean-substitution procedure, where mean subscale scores were substituted for missing values if only one item was missing. This resulted in 429 participants with complete scores on the body-related distress subscale (*M* = 37.73, *SD* = 19.36) and 438 participants with complete scores of the gender expression burden subscale (*M* = 26.89, *SD* = 14.91).

### 4.5 Reliability analyses

Reliability analyses, including recalculations with each item deleted, were conducted on the overall TASS-D scale and two subscales using Cronbach's alpha, via the *alpha* function from the *psych* package in R (Revelle, 2023), with 95% confidence intervals obtained through bootstrap estimation with 1,000 iterations using the *cronbach.alpha* function from the *ltm* package (Rizopoulos, 2006). No items were removed at this stage for weakening the overall scale or subscale reliabilities. Reliability of the overall scale was excellent [α = 0.914, 95% CI (0.903, 0.926)]. Reliability of the body-related distress subscale was high [α = 0.896, 95% CI (0.879, 0.909)], as was reliability of the gender expression burden subscale [α = 0.869, 95% CI (0.847, 0.887)]. For the whole scale, test-retest reliability at 2 weeks post baseline was *r* = 0.86 [95% CI: (0.82, 0.89)]. Test-retest reliability of the body-related distress subscale was *r* = 0.87 [95% CI: (0.84, 0.90)], and test-retest reliability of the gender expression burden subscale was *r* = 0.78 [95% CI: (0.72, 0.83)].

### 4.6 Convergent validity

Table 3 shows the correlations between TASS-D scores and the original UGDS, the UGDS-GS, and the GIDYQ-AA. TASS-D total and body-related distress scores were moderately positively correlated with the UGDS-F and UGDS-M forms. Correlations of these scales with the TASS-D gender expression burden subscale were weaker (UGDS-F: *r* = 0.37, *p* < 0.001; UGDS-M: *r* = 0.16, *p* = 0.31). Compared to the original UDGS forms, correlations of the TASS-D and subscales with the UGDS-GS scores were significant and positive, but smaller in magnitude. Correlations of the GIDYQ-AA scores with the TASS-D total score and body-related distress subscale score were negative and moderate. However, correlations with the gender expression burden subscale were not as strong.

**Table 3:** Correlations between TASS-D and subscales with other measures of gender dysphoria and gender minority stress.

**TASS-D lifetime**	**Whole scale**	**Body**	**Burden**
UGDS-F	0.59^*^	0.67^*^	0.37^*^
UGDS-M	0.43^*^	0.53^*^	0.16
UGDS-GS	0.24^*^	0.21^*^	0.19^*^
GIDYQ-AA-FAB	−0.37^*^	−0.43^*^	−0.20^*^
GIDYQ-AA-MAB	−0.41^*^	−0.50^*^	−0.18
SMASI lifetime	0.32^*^	0.28^*^	0.29^*^
GMSR discrimination	0.22^*^	0.31^*^	0.05
GMSR rejection	0.28^*^	0.23^*^	0.27^*^
GMSR victimization	0.26^*^	0.24^*^	0.22^*^
GMSR nonaffirmation	0.41^*^	0.26^*^	0.51^*^
GMSR internalized transphobia	0.36^*^	0.23^*^	0.44^*^
GMSR pride	−0.35^*^	−0.33^*^	−0.29^*^
GMSR negative expectancies, Form A	0.26^*^	0.21	0.27^*^
GMSR negative expectancies, Form B	0.34^*^	0.22^*^	0.45^*^
GMSR non-disclosure, Form A	0.34^*^	0.40^*^	0.14
GMSR non-disclosure, Form B	0.62^*^	0.61^*^	0.51^*^
GMSR community connectedness	0.09	0.09	0.08

UGDS and GIDYQ scales with -F or -M markers were administered to participants assigned female or male at birth, respectively. GMSR Form A was administered to those not living in their affirmed gender; Form B was administered to those currently living in their affirmed gender.

^*^Indicates correlations significant at *p* < 0.05.

### 4.7 Divergent validity

Table 3 also presents the correlations between the TASS-D and the individual scales of the GMSR, which measures various constructs related to gender minority stress and resilience. Most negative GMSR subscales showed positive but weak correlations (*r* < 0.30) with the TASS-D and its subscales. Pride was weakly negatively correlated with all aspects of the TASS-D, whereas community connectedness showed no relationship to our measure of dysphoria. Of note, the GMSR Nondisclosure form for people currently living as their affirmed gender showed higher positive correlations with the TASS-D and its subscales—possibly because many of the items in this GMSR subscale involve modifying or monitoring gender expression to conceal gender history. Similarly, the correlations of the TASS-D and the SMASI, another measure of minority stress, were positive but weak *(r* = 0.32, *p* < 0.001 for the whole TASS-D; *r* = 0.28–0.29 for the TASS-D subscales).

### 4.8 Criterion validity

To assess criterion validity, we analyzed the associations between the TASS-D total scale and the two TASS-D subscales with mental health and behavioral outcomes. For continuous outcomes, we ran multiple regression models, regressing the outcome with the TASS-D (or subscale), controlling for the same demographic features we included in the measurement invariance analyses. For binary outcomes, we ran logistic regression models using the same predictors. Table 4 provides the standardized regression estimates (for continuous outcomes) or odds ratios (for binary outcomes) associated with the TASS-D or subscale score for every outcome. Every entry in Table 4 represents a separate analysis that included demographic control variables (full supplemental results available on request).

**Table 4:** Standardized regression estimates and odds ratios associated with the TASS-D and subscales.

**Outcome**	**TASS-D**	**TASS-D body subscale**	**TASS-D burden subscale**
**Continuous outcomes**			
Depression (CES-D-4)	0.39^*^	0.30^*^	0.42^*^
Anxiety (GAD-7)	0.42^*^	0.35^*^	0.39^*^
PTSD (PCL-C-6)	0.45^*^	0.35^*^	0.45^*^
**Suicidality and self-harm**			
Suicidal ideation	1.02^*^	1.02^*^	1.04^*^
Suicide attempt	1.02^*^	1.02^*^	1.04^*^
Non-suicidal self-injury	1.01^*^	1.02^*^	1.03^*^
**Alcohol use**			
Past 30-day	1.00	1.00	0.99
Lifetime	1.00	1.00	0.99
**Tobacco use**			
Past 30-day	0.99	0.99	0.99
Lifetime	1.00	1.00	1.00
**Marijuana use**			
Past 30-day	1.01	1.01	1.02^*^
Lifetime	1.01^*^	1.02^*^	1.03^*^
**Prescription drug use**			
Past 30-day	1.02^*^	1.03	1.02
Lifetime	0.99	1.00	0.99
**Illicit drug use**			
Past 30-day	*NA*	*NA*	*NA*
Lifetime	1.00	1.02	1.00

Parameter estimates associated with the TASS-D and its subscales are taken from multiple regression (for continuous outcomes) and logistic regressions (for binary outcomes) that control for demographic features. Standardized regression parameters are reported for continuous outcomes, odds ratios are reported for binary outcomes. Parameters significant at p <0.05 are indicated by ^*^.

Outcomes where the estimate is reported as NA had insufficient occurrence in the data to estimate the model.

#### 4.8.1 Depressive symptoms

TASS-D total scores were positively associated with CES-D-4 scores (β = 0.39, *p* < 0.001). TASS-D body-related distress scores were also significantly associated with higher CES-D-4 scores (β = 0.30, *p* < 0.001), as were TASS-D gender expression burden scores (β = 0.42, *p* < 0.001).

#### 4.8.2 Anxiety symptoms

TASS-D total scores were positively associated with GAD-7 scores (β = 0.42, p <0.001). TASS-D body-related distress scores were also significantly associated with higher GAD-7 scores (β = 0.35, *p* < 0.001), as were TASS-D gender expression burden scores (β = 0.39, *p* < 0.001).

#### 4.8.3 PTSD symptoms

TASS-D total scores were positively associated with PCL-C-6 scores (β = 0.45, *p* < 0.001). TASS-D body-related distress scores (β = 0.35, *p* < 0.001) and TASS-D gender expression burden scores (β = 0.45, *p* < 0.001) were also significantly associated with higher PCL-C-6 scores.

#### 4.8.4 Suicidality and nonsuicidal self-injury

TASS-D totals scores were associated with slightly elevated odds of suicidal ideation [*OR* = 1.02, 95% CI (1.01, 1.03)], suicide attempt [*OR* = 1.02, 95% CI (1.01, 1.03)], and nonsuicidal self-injury [*OR* = 1.01, 95% CI (1.01, 1.02)]. For the body-related distress subscale, we also found elevated odds of suicidal ideation [*OR* = 1.02, 95% CI (1.01, 1.04)], suicide attempt [*OR* = 1.02, 95% CI (1.01, 1.04)], and nonsuicidal self-injury [*OR* = 1.02, 95% CI (1.00, 1.03)]. For the gender expression burden subscale, we found elevated odds of suicidal ideation [*OR* = 1.04, 95% CI (1.02, 1.05)], suicide attempt [*OR* = 1.04, 95% CI (1.02, 1.06)], and nonsuicidal self-injury [*OR* = 1.03, 95% CI (1.01, 1.04)], which were all slightly higher than the odds associated with the TASS-D total score and body-related distress subscale score.

#### 4.8.5 Substance use

##### 4.8.5.1 Alcohol

Total TASS-D scores were not associated with the odds of past-30-day alcohol use [*OR* = 1.00, 95% CI (0.99, 1.01)] or lifetime alcohol use [*OR* = 1.00, 95% CI (0.99, 1.01)]. TASS-D body-related distress subscale scores were also not related to the odds of alcohol use in the past 30 days [*OR* = 1.00, 95% CI (0.99, 1.02)] or lifetime [*OR* = 1.00, 95% CI (0.99, 1.01)]. The same findings were true for TASS-D gender expression burden subscale scores [past-30-day use: *OR* = 0.99, 95% CI (0.98, 1.01); lifetime use: *OR* = 0.99, 95% CI (0.98, 1.01)].

##### 4.8.5.2 Tobacco

TASS-D total scores were not associated with the odds of past-30-day tobacco use [*OR* = 0.99, 95% CI (0.98, 1.01)] or lifetime tobacco use [*OR* = 1.00, 95% CI (0.99, 1.01)]. This was also true for the TASS-D body-related distress subscale [past-30-day use: *OR* = 0.99, 95% CI (0.98, 1.01); lifetime use: *OR* = 0.99, 95% CI (0.97, 1.01)] and the TASS-D gender expression burden subscale [past-30-day use: *OR* = 0.99, 95% CI (0.97, 1.01); lifetime use: *OR* = 1.00, 95% CI (0.98, 1.02)].

##### 4.8.5.3 Marijuana

TASS-D total scores were not associated with the odds of past-30-day marijuana use [*OR* = 1.01, 95% CI (0.99, 1.02)], but were associated with slightly elevated odds of lifetime use [*OR* = 1.01, 95% CI (1.00, 1.02)]. Similarly, TASS-D body-related distress subscale scores were associated with higher odds of lifetime marijuana use [*OR* = 1.02, 95% CI (1.00, 1.03)] but not past-30-day use [*OR* = 1.01, 95% CI (0.99, 1.02)]. TASS-D gender expression burden subscale scores were associated with higher odds of both past-30-day use [*OR* = 1.02, 95% CI (1.00, 1.05)] and lifetime use [*OR* = 1.03, 95% CI (1.01, 1.04)].

##### 4.8.5.4 Prescription drugs

Prescription drug use included the use of pain relievers, prescription stimulants, and tranquilizers. TASS-D total scores were associated with slightly higher odds of past-30-day prescription drug use [*OR* = 1.02, 95% CI (1.00, 1.04)], but not lifetime use [*OR* = 0.99, 95% CI (0.99, 1.01)]. TASS-D body-related distress subscale scores were also associated with higher odds of past-30-day prescription drug use [*OR* = 1.03, 95% CI (1.00, 1.07)], but not lifetime use [*OR* = 1.00, 95% CI (0.99, 1.02)]. TASS-D gender expression burden scores were not associated with odds of past-30-day use [*OR* = 1.02, 95% CI (0.99, 1.08)] or lifetime use [*OR* = 0.99, 95% CI (0.97, 1.02)].

##### 4.8.5.5 Illicit drugs

Illicit drugs included cocaine, heroin, fentanyl, and methamphetamine. Use of any of these substances was exceedingly rare in our sample; only one case existed of past = 30-day use, so logistic regressions could not be run. Odds of lifetime illicit drug use were not associated with total TASS-D scores [*OR* = 1.01, 95% CI (0.98, 1.04)], TASS-D body-related distress scores [*OR* = 1.02, 95% CI (0.99, 1.02)], or TASS-D gender expression burden scores [*OR* = 1.00, 95% CI (0.95, 1.06)].

## 5 Discussion

The current study sought to address the need for an updated and psychometrically sound measure of gender dysphoria that is answerable by varied groups of transgender, nonbinary, and other gender-diverse and gender-expansive adolescents. Life history interviews with TNBA formed the basis for the development of candidate items, which were then subjected to expert panel review through a modified Delphi process, cognitive interviews with TNBA, and quantitative testing that incorporated factor analytic, IRT, and conventional reliability and validity assessment approaches. The final measure, the Transgender Adolescent Stress Survey-Dysphoria or TASS-D, provides a statistically robust and valid option for assessment of gender dysphoria that has been tested and performs well with an inclusive sample of TNBA. The final measure incorporates two subscales, representing body-related distress and gender expression burden. Statements of bodily incongruence (“The size of my chest does not match my gender identity”) compose the body-related distress subscale, whereas items composing the gender expression burden subscale (“It takes a lot of effort to express my gender identity”) reflect the cognitive energy spent managing and concealing incongruence between the respondents' gender identity and body.

Study results confirm the hypothesized relationships between the TASS-D and several existing measures of gender dysphoria and gender minority stress, establishing support for its convergent and divergent validity, respectively. In other words, this measure is statistically specific and relevant to the construct of gender dysphoria. Responses were stable over time, such that the scale benefits from high test–retest reliability, with benefits to future cross-sectional and longitudinal research alike. Finally, the TASS-D accounts for notable variance in mental health outcomes, including depression (*R*^2^ = 0.18), anxiety (*R*^2^ = 0.19), and PTSD (*R*^2^ = 0.19). The TASS-D was not associated with most substance use outcomes, but all substance use was relatively low, and use of illicit and prescription drugs was reported so rarely in the study population that these findings may be attributable to low statistical power. In sum, the TASS-D is a valid measure of gender dysphoria and showed the expected strong positive associations with several measures of psychological distress.

The TASS-D provides several important benefits in the assessment of gender dysphoria. The measure is answerable by binary and nonbinary transgender adolescents, alike, primarily because the measure does not presume that youth are transitioning or have a desire to transition from one gender to a purported “opposite” or other gender, nor that medical gender transition is an end goal for respondents. Prior scales of gender dysphoria have used sex assigned at birth as an organizing principle for measurement—for example, the GIDYQ-AA (Deogracias et al., 2007) has separate forms for respondents assigned male vs. female at birth and items assume binary transition goals (e.g., respondents assigned female at birth are asked “In the past 12 months, have you felt satisfied being a man?”). This may explain the negative correlations between the TASS-D and both forms of the GIDYQ-AA, an initially surprising conceptual result that may be better understood when one considers that the items of the GIDYQ-AA are not answerable by nonbinary or agender people who were present in our sample. The TASS-D consists of one form that is feasible for use by all respondents, thus serving as a broadly useful tool for measurement of gender dysphoria.

The TASS-D also asks separately about exposure to and distress from each experience, such that the measure does not presume that experiencing incongruence (e.g., “My genitals do not match my gender identity”) is necessarily a dysphoric experience for all transgender and nonbinary people. For each item, respondents endorse whether they experience that specific form of incongruence; if they do, then they are prompted to identify how distressing that experience is for them, if at all. As such, a person can describe their experience of their body in relation to their gender without it being assumed that that experience was distressing. Finally, the dysphoria measurement is continuous and recognizes that distress severity can vary across individuals and experiences of incongruence. In fact, although invariance testing established that the TASS-D exhibits configural and metric invariance, transmasculine respondents appeared to report higher levels of dysphoric distress than other respondents. Scalar variance by gender identity, sex assigned at birth, sexual identity, and pubertal development or use of hormones or blockers may point to true population differences in dysphoria distress or population differences in reporting of distress; future research may investigate subpopulation differences. The design of the TASS-D with a continuous scale of dysphoric symptom severity, rather than categorically determining whether participants meet clinical criteria for dysphoria, encourages further fine-tuned examination of such population differences.

Measure development of the TASS-D was conducted concurrently with the TASS-MS measure of gender minority stress. Based on a review of prior measures of minority stress and dysphoria (used in this study for convergent and divergent validation) and recommendations of the expert panel, the study team intentionally sought to produce distinct measures of dysphoria and minority stress. Recent scholarship has debated the construct specificity, interrelation, and overlap of dysphoria and minority stress, with the argument hinging on whether dysphoria inherently originates from social interaction (as minority stress does) or from the individual's experience of incongruence between self and body (Galupo et al., 2021; Lindley and Galupo, 2020). Although some recent work has successfully modeled dysphoria as a factor of the minority stress construct (Lindley and Galupo, 2020), that analysis relied on measures that pool dysphoria and minority stress items. The study team's decision to produce separate measures of dysphoria and minority stress allows future researchers to test whether these constructs are wholly distinct from each other.

Design of a measure of gender dysphoria must necessarily contend with long-standing debates on the medicalization of TNBA experiences and the real medical barriers to transition care created by diagnostic criteria, medical gatekeepers, and trans-antagonistic policymakers. The TASS-D, which was developed based on life history interviews with TNBA about their personal lived experiences of dysphoria, was not designed to capture DSM-5 gender dysphoria clinical criteria and thus was not intended to be a clinical diagnostic measure of gender dysphoria. Instead, the TASS-D uses a continuous scale with no clinical cutoff points above or below which a person would be deemed to have clinical levels of gender dysphoria. Notably, the TASS-D avoids assuming binary gender identification and transition goals, thereby offering an inclusive nonclinical means of understanding adolescent gender dysphoria. With that said, the TASS-D may have some future value in clinical settings. Notably, TNBA face substantial barriers to accessing and receiving gender-affirming medical and mental health care (Clark et al., 2018b; Cruz, 2014; Gridley et al., 2016; Schulz, 2018), and existing clinical measures of gender dysphoria used with TNBA are not inclusive of all genders, are not developmentally specific, and lack validity. Although the TASS-D is not designed for clinical diagnosis, future research may investigate its utility in assessing dysphoria symptom severity and the effects of interventions on symptom severity in important social contexts, including clinical settings.

Study results and future use of the TASS-D should be considered in light of study limitations. Although the TASS-D is intended for use with general populations of TNBA regardless of clinical engagement, original candidate items were composed using life history interviews with TNBA who were already using blockers or hormones. Although it is possible that highly unique experiences of nonclinically engaged TNBA may not have been included in original candidate items, the Delphi process and subsequent cognitive interviews with TNBA (not all of whom were clinically engaged) each resulted in the addition of items that may have addressed this gap. Additionally, the life history interview process captured adolescents' memories of experiences with incongruence beginning prior to initiating trans-affirming care and up to current experiences after starting blockers or hormones or both, such that their experiences may reflect broader populations of TNBA. Statistical analyses had sufficient statistical power and groups were adequately represented to test for invariance; however, the sample was predominantly assigned female at birth (73.6%), White (65.5%), and living in an urban setting (82.7%). This sample reflects those found in other nationwide studies (Salk et al., 2020) and may reflect challenges in research recruitment specific to TNBA subgroups. Future research may investigate scalar invariance by gender identity, sex assigned at birth, sexual identity, and pubertal development; study findings of scalar variance by pubertal development may indicate the need to include account for its variance in future research with the TASS-D.

This study developed and validated a novel measure of gender dysphoria, the TASS-D. The measure offers notable benefits over existing measures: It is psychometrically sound, inclusive of all gender identities, and does not assume that respondents identify with a binary gender or have medical transition as a terminal goal. Recent study findings that medical transition may relieve gender dysphoria for some and present “a new source of distress” (Galupo et al., 2021, p. 108) for other nonbinary and agender participants underscore the benefit of dysphoria measures that do not assume transition goals. The scale allows TNBA to endorse experiences of incongruence between their gender and body and then separately measures whether this incongruity is distressing, reflecting adolescents' complex lived experiences of gender embodiment. Thus, as previous studies have also emphasized, distress is not a necessary characteristic of being trans or a defining condition of incongruence (Chen et al., 2016; Galupo et al., 2021). Moreover, this design allows measurement of whether persisting incongruity (a respondent who repeatedly endorses that “My body does not match my gender identity”) may become more or less distressing over time. The scale is continuous, allowing measurement of symptom severity and conferring potential clinical utility of understanding individual and population experiences of distress. The scale is not designed to determine diagnostically whether a person should be provided or denied intervention; rather it can be a salient tool to understand incongruence and distress across important social, developmental, and temporal contexts of life.

Given some scalar non-invariance by gender identity, sex assigned at birth, sexual identity, and pubertal development and use of hormones or blockers, future research may test population or contextual differences in dysphoria severity. As an inclusive measure, the TASS-D provides opportunities to examine nonbinary/agender dysphoria experiences quantitatively and possibly inform future intervention, both of which constitute ongoing gaps in the literature. Most importantly and as suggested by recent studies (Galupo et al., 2021; Lindley and Galupo, 2020), future research may use the TASS-D to understand dysphoria as it is experienced longitudinally throughout adolescence and how experiences of gender dysphoria may shift at individual and community levels in response to sociopolitical change; social, mental, and medical health interventions; and in developmentally specific contexts such as family and school.

## Data Availability

The raw data supporting the conclusions of this article will be made available by the authors, without undue reservation.

## Appendix A

**Table A1:** Transgender Adolescent Stress Scale-Dysphoria (TASS-D).

**Original item #**	**Item text**
**TASS-D: Body related distress (7 items)**	
2	My genitals do not match my gender identity
3	The size of my chest does not match my gender identity
7	The way my body experiences sexual arousal feels wrong to me
8	My body does not match my gender identity
9	My body is developing in ways that feel wrong for my gender identity
17	I try to hide parts of my body because they do not match my gender identity
18	I try to avoid seeing parts of my body that do not match my gender identity
**TASS-D: Gender Expression Burden (5 items)**	
14	My body will never fully match my gender
15	Making my gender expression match my gender will be a burden for the rest of my life
16	I worry I will never be seen as my gender identity
19	Making my appearance match my gender identity is exhausting
20	It takes a lot of effort to express my gender identity
